# Crystal structure and DFT study of 8-hy­droxy-1,2,3,5,6,7-hexa­hydro­pyrido[3,2,1-*ij*]quinoline-9-carbaldehyde

**DOI:** 10.1107/S2056989017005886

**Published:** 2017-04-28

**Authors:** Md. Serajul Haque Faizi, Necmi Dege, Maria L. Malysheva

**Affiliations:** a Department of Chemistry, College of Science, Sultan Qaboos University, PO Box 36 Al-Khod 123, Muscat, Oman; b Ondokuz Mayıs University, Arts and Sciences Faculty, Department of Physics, 55139 Samsun, Turkey; c Department of Chemistry, Taras Shevchenko National University of Kyiv, 64, Vladimirska Str., Kiev 01601, Ukraine

**Keywords:** crystal structure, 8-hy­droxy­julolidine, julolidine, hydrogen bonding

## Abstract

In the title compound, C_13_H_15_NO_2_, the fused non-aromatic rings of the julolidine moiety adopt envelope conformations. The hy­droxy group forms an intra­molecular hydrogen bond to the aldehyde O atom, generating an *S*(6) ring motif. Weak inter­molecular C—H⋯O hydrogen bonds help to stabilize the crystal structure. Density functional theory (DFT) optimized structures at the B3LYP/6–311 G(d,p) level are compared with the experimentally determined mol­ecular structure in the solid state.

## Abstract 2

In the title compound, the hy­droxy group forms an intra­molecular hydrogen bond to the aldehyde O atom, generating an *S*(6) ring motif. The fused non-aromatic rings of the julolidine moiety adopt envelope conformations. Geometrical parameters, determined using X-ray diffraction techniques, are compared with those calculated by density functional theory (DFT), using the B3LYP/6–311 G(d,p) level of theory.

## Chemical context   

Julolidine is chemically an aniline derivative with two *N*-alkyl substituents forming rings back to the aromatic ring; the fused rings lock the nitro­gen lone-pair of electrons into conjugation with the aromatic ring leading to unusual reactivity. The presence of the julolidine ring system in some mol­ecules makes them useful for chromogenic naked-eye detection of copper, zinc, iron and aluminium ions as well as fluoride ions (Wang *et al.*, 2013 ▸; Choi *et al.*, 2015 ▸; Kim *et al.*, 2015 ▸; Jo *et al.*, 2015 ▸). Julolidine dyes exhibit excited-state intra­molecular proton transfer (Nano *et al.*, 2015 ▸). Compounds containing lulolidine rings are also used as fluorescent probes for the measurement of cell-membrane viscosity. Julolidine-based materials are also used as red emitters in OLEDs when linked to di­cyano­methyl­pyran modules (Lee, *et al.*, 2012 ▸). The julol­idine unit plays an important role as it has strong electronic-donating properties for chelating (Nano, *et al.*, 2013 ▸). Julol­idine malono­nitrile acts as a ‘push–pull’ mol­ecule with large hyperpolarizability and is used as a model system for understanding the non-linear optical properties of mol­ecules (Mennucci *et al.*, 2009 ▸).

There are many reports in the literature on julolidine-based Schiff bases and their applications as sensors for metal ions (Park *et al.*, 2014 ▸; Lee *et al.*, 2014 ▸; Kim *et al.*, 2016 ▸). The present work is a part of an ongoing structural study of Schiff bases based on the julolidine ring system (Faizi *et al.*, 2016 ▸, 2017 ▸). We report here the crystal structure and DFT computational calculation of the title julolidine compound (I). The results of calculations by density functional theory (DFT) on (I) carried out at the B3LYP/6–311 G(d,p) level are compared with the experimentally determined mol­ecular structure in the solid state.
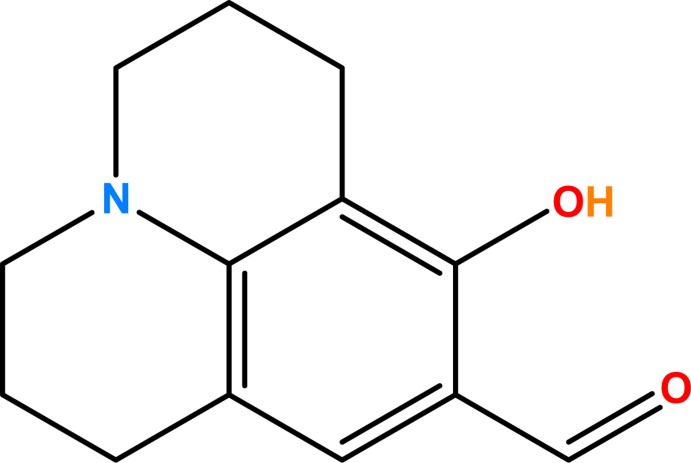


## Structural commentary   

**Figure 1:**
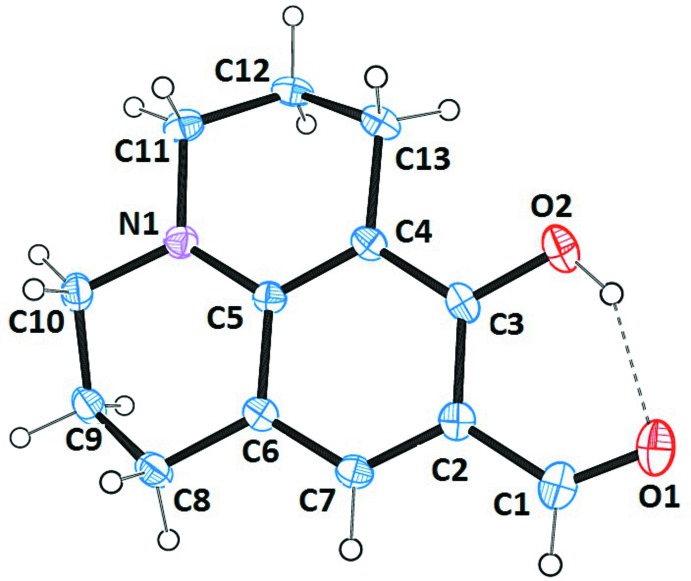
The mol­ecular structure of the title compound, with the atom labelling. Displacement ellipsoids are drawn at the 30% probability level. The intra­molecular O—H⋯O hydrogen bond is shown as a dashed line (see Table 1 ▸).

The mol­ecular structure of the title compound (I) is shown in Fig. 1 ▸. The π-conjugated system is nearly planar, with a 2.5 (1)° twist between the aromatic and aldehyde groups. The julol­idine ring system comprises three fused rings and one locked nitro­gen atom. The C1—O1 and C3—O2 bond lengths are of 1.231 (3) and 1.345 (3) Å, respectively, indicate double- and single-bond character for these bonds. The two fused non-aromatic rings of the julolidine moiety adopt slightly distorted envelope conformations with atoms C9 and C12 displaced from the plane through the remaining ring atoms by 0.654 (2) and 0.648 (2) Å, respectively. The intra­molecular O2—H2⋯O1 hydrogen bond forms an *S*(6) ring motif (Fig. 1 ▸ and Table 1 ▸) between the phenol and aldehyde groups. Such an intra­molecular hydrogen bond is common in salicyl­aldehyde derivatives, and the metrical parameters are comparable to those for related structures such as hy­droxy­benzaldehyde (Kirchner *et al.*, 2011 ▸).

## Supra­molecular features   

**Figure 2:**
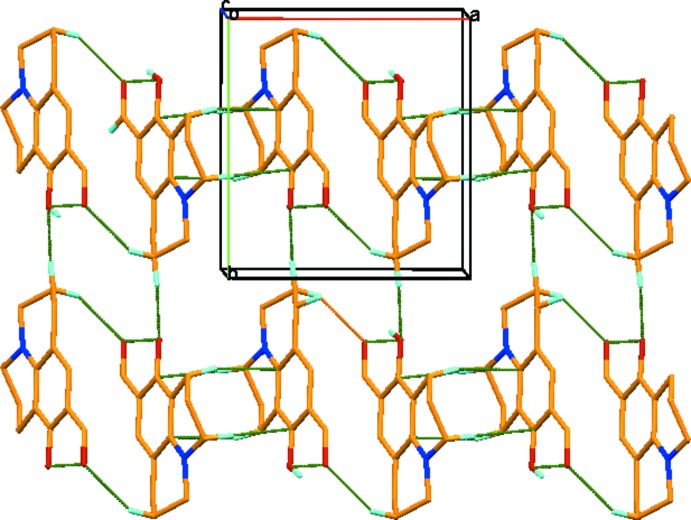
A view of the *A*–*B*–*A*–*B*–*A*–*B* arrangement in the crystal structure of the title compound. The hydrogen bonds are shown as dashed lines (see Table 1 ▸). For clarity, only the H atoms involved in hydrogen bonding have been included. The packing structure exhibits 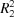
(16) and 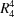
(10) graph-set motifs.

In the crystal, mol­ecules are linked by C—H⋯O hydrogen bonds, forming an *A*–*B-*-*A*–*B*–*A*–*B* arrangement through the inversion centre and propagating along the *c*-axis direction (see Fig. 2 ▸ and Table 1 ▸). There are no other significant inter­molecular contacts present in the mol­ecule.

## DFT study   

The DFT quantum-chemical calculations were performed at the B3LYP/6–311 G(d,p) level (Becke, 1993 ▸; Lee *et al.*, 1988 ▸) as implemented in *GAUSSIAN09* (Frisch *et al.*, 2009 ▸). DFT structure optimization of (I) was performed starting from the X-ray geometry and the values compared with experimental values (see Table 2 ▸). From these results we can conclude that basis set 6–311 G(d,p) is well suited in its approach to the experimental data.

**Figure 3:**
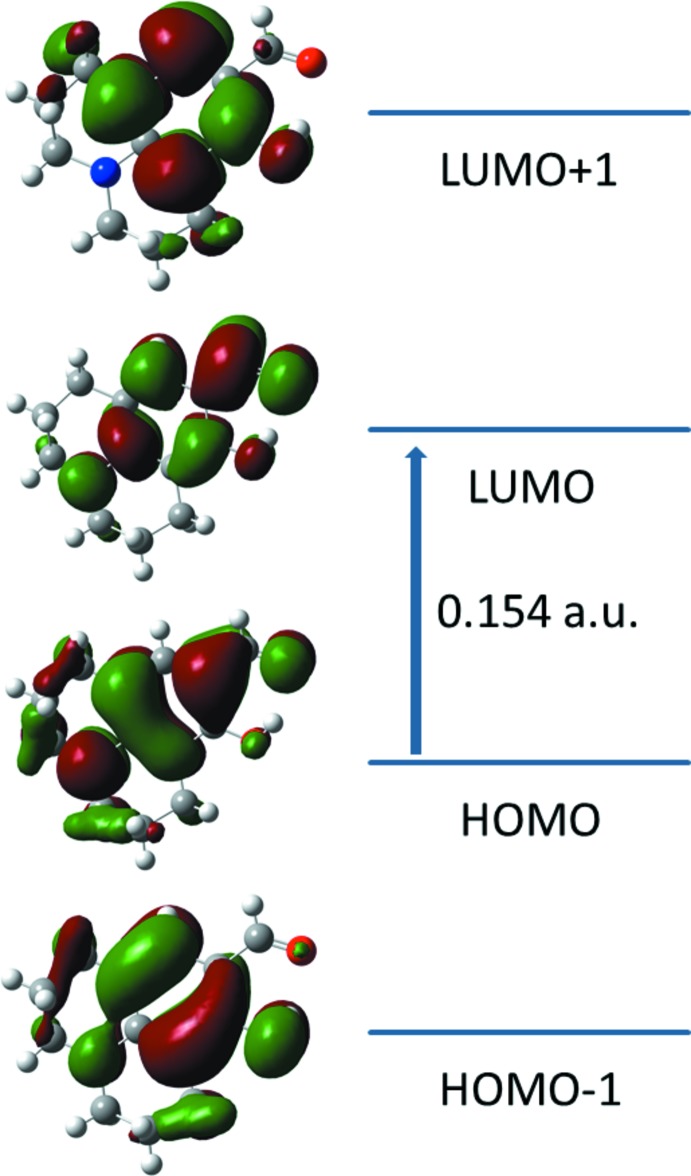
Electron distribution of the HOMO-1, HOMO, LUMO and the LUMO+1 energy levels for (I).

The DFT study of (I) shows that the HOMO and LUMO are localized in the plane extending from the whole julolidine ring to the salicyl­aldehyde ring. The electron distribution of the HOMO-1, HOMO, LUMO and the LUMO+1 energy levels are shown in Fig. 3 ▸. The mol­ecular orbital of HOMO contain both σ and π character whereas HOMO-1 is dominated by π-orbital density. The LUMO is mainly composed of σ density while LUMO+1 has both σ and π electronic density. The HOMO–LUMO gap was found to be 0.154 a.u. and the frontier mol­ecular orbital energies, *E*_HOMO_ and *E*_LUMO_ were f −0.19624 and −0.04201 a.u., respectively.

## Database survey   

A search of the Cambridge Structural Database (CSD, Version 5.37, update May 2016; Groom *et al.*, 2016 ▸) gave 121 hits for the julolidine moiety. Of these, six have an OH group in position 8, and four also have a C=N group in position 1. The very similar compound 2-[(2,3,6,7-tetra­hydro-1*H*,5*H*-benzo[*ij*]-quinolizin-9-yl)methyl­ene]propanedi­nitrile (II) reported by Liang *et al.* (2009 ▸) has the aldehydic group in (I) replaced by di­cyano­vinyl groups and the hy­droxy group replaced by hydrogen. The N1—C5 bond length [1.381 (2) Å] in the title compound is longer than in (II) [1.365 (3) Å] due to conjugation with di­cyano­vinyl group. In the julolidine-1,6-dione compound reported by Wu *et al.* (2007 ▸), the N atom of the julolidine moiety lies approximately in the plane of the benzene ring with a deviation of 0.023 (2) Å, similar to that in title compound [0.043 (2) Å], as might be expected for the maximum conjugation normally found for N-atom substit­uents on benzene rings.

## Crystallization   

2,3,6,7-Tetra­hydro-8-hy­droxy-1*H*,5*H*-benzo[*ij*]quinolizine-9-carboxaldehyde was purchased from Sigma Aldrich and crystallized by slow evaporation of methanol solution over a period of 2-3 days to yield quality crystal suitable for X-ray data collection.

## Refinement   

Crystal data, data collection and structure refinement details are summarized in Table 3 ▸. All H atoms were located from difference-Fourier maps but in the final cycles of refinement they were included in calculated positions and treated as riding atoms: O—H = 0.84 Å, C—H = 0.93–0.98 Å with *U*_iso_(H) = 1.5*U*_eq_(O) and 1.2*U*_eq_(C) for other H atoms.

## Supplementary Material

Crystal structure: contains datablock(s) I. DOI: 10.1107/S2056989017005886/hg5485sup1.cif

Structure factors: contains datablock(s) I. DOI: 10.1107/S2056989017005886/hg5485Isup2.hkl

Supporting information file. DOI: 10.1107/S2056989017005886/hg5485Isup3.cml

CCDC reference: 1533792

Additional supporting information:  crystallographic information; 3D view; checkCIF report

## Figures and Tables

**Table 1:** Hydrogen-bond geometry (Å, °)

*D*—H⋯*A*	*D*—H	H⋯*A*	*D*⋯*A*	*D*—H⋯*A*
O2—H2⋯O1	0.82	1.89	2.621 (2)	148
C9—H9*A*⋯O2^i^	0.97	2.50	3.324 (3)	143
C9—H9*B*⋯O1^ii^	0.97	2.55	3.438 (3)	152
O2—H2⋯O1	0.82	1.89	2.621 (2)	148
C9—H9*A*⋯O2^i^	0.97	2.50	3.324 (3)	143
C9—H9*B*⋯O1^ii^	0.97	2.55	3.438 (3)	152

Symmetry codes: (i) 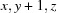; (ii) 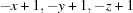.

**Table 2:** Comparison of selected geometric data for (I) (Å, °) from calculated (DFT) and X-ray data

Bonds	X-ray	B3LYP/6–311G(d,p)
C1—O1	1.231 (3)	1.231
C3—O2	1.345 (3)	1.345
C1—C2	1.431 (3)	1.431
N1—C5	1.381 (2)	1.381
O1—C1—C2	126.2 (2)	126.22
C1—C2—C3	121.34 (18)	120.25
C11—N1—C10	116.83 (15)	116.81

**Table 3:** Experimental details

Crystal data	
Chemical formula	C_13_H_15_NO_2_
*M*_r_	217.26
Crystal system, space group	Monoclinic, *P*2_1_/*c*
Temperature (K)	100
*a*, *b*, *c* (Å)	8.546 (3), 9.137 (3), 13.662 (4)
β (°)	95.984 (6)
*V* (Å^3^)	1061.0 (6)
*Z*	4
Radiation type	Mo *K*α
μ (mm^−1^)	0.09
Crystal size (mm)	0.18 × 0.15 × 0.11

Data collection	
Diffractometer	Bruker SMART CCD area detector
Absorption correction	Multi-scan (*SADABS*; Sheldrick, 2004 ▸)
*T*_min_, *T*_max_	0.985, 0.991
No. of measured, independent and observed [*I* > 2σ(*I*)] reflections	5787, 2083, 1530
*R*_int_	0.026
(sin θ/λ)_max_ (Å^−1^)	0.617

Refinement	
*R*[*F* ^2^ > 2σ(*F* ^2^)], *wR*(*F* ^2^), *S*	0.066, 0.231, 1.11
No. of reflections	2083
No. of parameters	145
H-atom treatment	H-atom parameters constrained
Δρ_max_, Δρ_min_ (e Å^−3^)	0.64, −0.27

Computer programs: *SMART* and *SAINT* (Bruker, 2003 ▸), *SHELXTL* and *SHELXL97* (Sheldrick, 2008 ▸).

## supplementary crystallographic information

### Crystal data

**Table:**

C_13_H_15_NO_2_	*F*(000) = 464
*M**_r_* = 217.26	*D*_x_ = 1.360 Mg m^−^^3^
Monoclinic, *P*2_1_/*c*	Mo *K*α radiation, λ = 0.71073 Å
*a* = 8.546 (3) Å	Cell parameters from 1494 reflections
*b* = 9.137 (3) Å	θ = 2.4–28.1°
*c* = 13.662 (4) Å	µ = 0.09 mm^−^^1^
β = 95.984 (6)°	*T* = 100 K
*V* = 1061.0 (6) Å^3^	Needle, colorless
*Z* = 4	0.18 × 0.15 × 0.11 mm

### Data collection

**Table:**

Bruker SMART CCD area detector diffractometer	2083 independent reflections
Radiation source: sealed tube	1530 reflections with *I* > 2σ(*I*)
Graphite monochromator	*R*_int_ = 0.026
phi and ω scans	θ_max_ = 26.0°, θ_min_ = 2.4°
Absorption correction: multi-scan (SADABS; Sheldrick, 2004)	*h* = −10→10
*T*_min_ = 0.985, *T*_max_ = 0.991	*k* = −8→11
5787 measured reflections	*l* = −16→16

### Refinement

**Table:**

Refinement on *F*^2^	0 restraints
Least-squares matrix: full	Hydrogen site location: inferred from neighbouring sites
*R*[*F*^2^ > 2σ(*F*^2^)] = 0.066	H-atom parameters constrained
*wR*(*F*^2^) = 0.231	*w* = 1/[σ^2^(*F*_o_^2^) + (0.1465*P*)^2^ + 0.0857*P*] where *P* = (*F*_o_^2^ + 2*F*_c_^2^)/3
*S* = 1.11	(Δ/σ)_max_ < 0.001
2083 reflections	Δρ_max_ = 0.64 e Å^−^^3^
145 parameters	Δρ_min_ = −0.27 e Å^−^^3^

### Special details

**Table:**

Geometry. All esds (except the esd in the dihedral angle between two l.s. planes) are estimated using the full covariance matrix. The cell esds are taken into account individually in the estimation of esds in distances, angles and torsion angles; correlations between esds in cell parameters are only used when they are defined by crystal symmetry. An approximate (isotropic) treatment of cell esds is used for estimating esds involving l.s. planes.

### Fractional atomic coordinates and isotropic or equivalent isotropic displacement parameters (Å^2^)

**Table:**

	*x*	*y*	*z*	*U*_iso_*/*U*_eq_	
O2	0.73084 (18)	0.25645 (16)	0.53899 (11)	0.0398 (5)	
H2	0.691533	0.226583	0.587471	0.060*	
O1	0.58740 (18)	0.27075 (18)	0.69921 (11)	0.0460 (5)	
N1	0.83012 (18)	0.7058 (2)	0.37861 (11)	0.0288 (5)	
C5	0.7743 (2)	0.6300 (2)	0.45521 (12)	0.0242 (5)	
C4	0.7801 (2)	0.4768 (2)	0.45803 (13)	0.0269 (5)	
C6	0.7080 (2)	0.7115 (2)	0.53120 (14)	0.0277 (5)	
C2	0.6517 (2)	0.4816 (2)	0.60979 (14)	0.0282 (5)	
C7	0.6468 (2)	0.6333 (2)	0.60434 (14)	0.0282 (5)	
H7	0.599815	0.684412	0.652391	0.034*	
C3	0.7204 (2)	0.4032 (2)	0.53505 (14)	0.0282 (5)	
C10	0.8409 (2)	0.8646 (2)	0.37928 (14)	0.0333 (6)	
H10A	0.840281	0.899757	0.312243	0.040*	
H10B	0.939673	0.893952	0.415420	0.040*	
C11	0.9103 (2)	0.6288 (2)	0.30450 (15)	0.0330 (6)	
H11A	1.020944	0.618318	0.327631	0.040*	
H11B	0.902214	0.685601	0.244223	0.040*	
C13	0.8464 (2)	0.3888 (2)	0.37783 (14)	0.0336 (6)	
H13A	0.786287	0.299427	0.365792	0.040*	
H13B	0.954748	0.362366	0.398627	0.040*	
C8	0.7061 (3)	0.8758 (2)	0.52940 (15)	0.0359 (6)	
H8A	0.797720	0.912690	0.569753	0.043*	
H8B	0.613101	0.910814	0.557226	0.043*	
C1	0.5867 (2)	0.4043 (3)	0.68718 (15)	0.0358 (6)	
H1	0.539410	0.460267	0.732734	0.043*	
C12	0.8388 (2)	0.4791 (2)	0.28373 (14)	0.0330 (6)	
H12A	0.895351	0.428934	0.235680	0.040*	
H12B	0.730055	0.489689	0.256334	0.040*	
C9	0.7061 (3)	0.9339 (2)	0.42599 (15)	0.0389 (6)	
H9A	0.718103	1.039438	0.427433	0.047*	
H9B	0.607130	0.910495	0.387813	0.047*	

### Atomic displacement parameters (Å^2^)

**Table:**

	*U*^11^	*U*^22^	*U*^33^	*U*^12^	*U*^13^	*U*^23^
O2	0.0514 (11)	0.0255 (10)	0.0406 (9)	−0.0001 (6)	−0.0050 (7)	0.0021 (6)
O1	0.0469 (11)	0.0421 (11)	0.0470 (10)	−0.0075 (7)	−0.0054 (7)	0.0141 (7)
N1	0.0304 (9)	0.0301 (11)	0.0264 (9)	−0.0015 (7)	0.0044 (7)	−0.0016 (6)
C5	0.0233 (10)	0.0269 (13)	0.0217 (10)	−0.0009 (7)	−0.0011 (8)	−0.0033 (7)
C4	0.0289 (12)	0.0273 (12)	0.0233 (11)	0.0013 (7)	−0.0034 (8)	−0.0030 (7)
C6	0.0278 (11)	0.0275 (12)	0.0270 (10)	0.0021 (8)	−0.0007 (8)	−0.0016 (8)
C2	0.0263 (11)	0.0314 (13)	0.0256 (11)	−0.0007 (8)	−0.0041 (8)	0.0020 (8)
C7	0.0289 (11)	0.0307 (13)	0.0244 (10)	0.0010 (8)	−0.0005 (8)	−0.0043 (8)
C3	0.0286 (12)	0.0220 (11)	0.0319 (12)	0.0005 (7)	−0.0069 (9)	−0.0005 (8)
C10	0.0408 (13)	0.0294 (13)	0.0298 (11)	−0.0084 (8)	0.0032 (9)	0.0002 (8)
C11	0.0290 (11)	0.0437 (14)	0.0264 (11)	0.0013 (9)	0.0036 (8)	−0.0040 (9)
C13	0.0372 (13)	0.0295 (13)	0.0332 (12)	0.0069 (8)	−0.0002 (9)	−0.0071 (8)
C8	0.0484 (14)	0.0272 (13)	0.0323 (12)	0.0008 (9)	0.0050 (9)	−0.0049 (8)
C1	0.0291 (12)	0.0411 (14)	0.0353 (12)	−0.0037 (9)	−0.0051 (9)	0.0087 (9)
C12	0.0329 (12)	0.0389 (13)	0.0265 (11)	0.0085 (9)	0.0001 (8)	−0.0099 (8)
C9	0.0543 (14)	0.0252 (12)	0.0368 (12)	−0.0019 (9)	0.0028 (10)	−0.0026 (9)

### Geometric parameters (Å, º)

**Table:**

O2—C3	1.345 (3)	C10—H10A	0.9700
O2—H2	0.8200	C10—H10B	0.9700
O1—C1	1.231 (3)	C11—C12	1.512 (3)
N1—C5	1.381 (2)	C11—H11A	0.9700
N1—C10	1.454 (3)	C11—H11B	0.9700
N1—C11	1.461 (2)	C13—C12	1.523 (3)
C5—C4	1.401 (3)	C13—H13A	0.9700
C5—C6	1.441 (3)	C13—H13B	0.9700
C4—C3	1.390 (3)	C8—C9	1.509 (3)
C4—C13	1.516 (3)	C8—H8A	0.9700
C6—C7	1.376 (3)	C8—H8B	0.9700
C6—C8	1.502 (3)	C1—H1	0.9300
C2—C7	1.389 (3)	C12—H12A	0.9700
C2—C3	1.423 (3)	C12—H12B	0.9700
C2—C1	1.431 (3)	C9—H9A	0.9700
C7—H7	0.9300	C9—H9B	0.9700
C10—C9	1.513 (3)		
			
C3—O2—H2	109.5	N1—C11—H11B	109.5
C5—N1—C10	121.51 (16)	C12—C11—H11B	109.5
C5—N1—C11	120.49 (19)	H11A—C11—H11B	108.1
C10—N1—C11	116.83 (15)	C4—C13—C12	109.65 (17)
N1—C5—C4	120.54 (16)	C4—C13—H13A	109.7
N1—C5—C6	118.7 (2)	C12—C13—H13A	109.7
C4—C5—C6	120.79 (17)	C4—C13—H13B	109.7
C3—C4—C5	119.32 (17)	C12—C13—H13B	109.7
C3—C4—C13	119.00 (19)	H13A—C13—H13B	108.2
C5—C4—C13	121.66 (17)	C6—C8—C9	111.45 (16)
C7—C6—C5	117.6 (2)	C6—C8—H8A	109.3
C7—C6—C8	121.81 (17)	C9—C8—H8A	109.3
C5—C6—C8	120.62 (17)	C6—C8—H8B	109.3
C7—C2—C3	118.46 (18)	C9—C8—H8B	109.3
C7—C2—C1	121.34 (18)	H8A—C8—H8B	108.0
C3—C2—C1	120.2 (2)	O1—C1—C2	126.2 (2)
C6—C7—C2	123.07 (18)	O1—C1—H1	116.9
C6—C7—H7	118.5	C2—C1—H1	116.9
C2—C7—H7	118.5	C11—C12—C13	110.52 (16)
O2—C3—C4	119.02 (17)	C11—C12—H12A	109.5
O2—C3—C2	120.24 (18)	C13—C12—H12A	109.5
C4—C3—C2	120.7 (2)	C11—C12—H12B	109.5
N1—C10—C9	111.68 (16)	C13—C12—H12B	109.5
N1—C10—H10A	109.3	H12A—C12—H12B	108.1
C9—C10—H10A	109.3	C8—C9—C10	108.80 (19)
N1—C10—H10B	109.3	C8—C9—H9A	109.9
C9—C10—H10B	109.3	C10—C9—H9A	109.9
H10A—C10—H10B	107.9	C8—C9—H9B	109.9
N1—C11—C12	110.87 (16)	C10—C9—H9B	109.9
N1—C11—H11A	109.5	H9A—C9—H9B	108.3
C12—C11—H11A	109.5		

### Hydrogen-bond geometry (Å, º)

**Table:**

*D*—H···*A*	*D*—H	H···*A*	*D*···*A*	*D*—H···*A*
O2—H2···O1	0.82	1.89	2.621 (2)	148
C9—H9*A*···O2^i^	0.97	2.50	3.324 (3)	143
C9—H9*B*···O1^ii^	0.97	2.55	3.438 (3)	152
O2—H2···O1	0.82	1.89	2.621 (2)	148
C9—H9*A*···O2^i^	0.97	2.50	3.324 (3)	143
C9—H9*B*···O1^ii^	0.97	2.55	3.438 (3)	152

Symmetry codes: (i) *x*, *y*+1, *z*; (ii) −*x*+1, −*y*+1, −*z*+1.
